# Supplementary material for: A Novel Gene Synthesis Platform for Designing Functional Protein Polymers
Source: Adv Sci (Weinh). 2025 Feb 23;12(15):2410903. doi: 10.1002/advs.202410903 (PMC12005822; doi:10.1002/advs.202410903)
Supplement: Supplementary file 1 — Supporting Information [file ADVS-12-2410903-s001.docx]

Supporting Information

**A Novel Gene Synthesis Platform for Designing Functional Protein Polymers**

*Toshimasa Homma^*^, Rie Yamamoto, Lily Zuin Ping Ang, Alaa Fehaid, Mitsuhiro Ebara*

**Figure S1. Linear vector for protein polymer expression**

Red letters indicate overlapping sequences. Sequences at both ends of the linear vector were designed to add MK to the N-terminus and WPTHHHHHH to the C-terminus of the expressed protein polymers. Red and yellow arrows indicate the primer annealing positions for colony PCR and Sanger DNA sequencing.


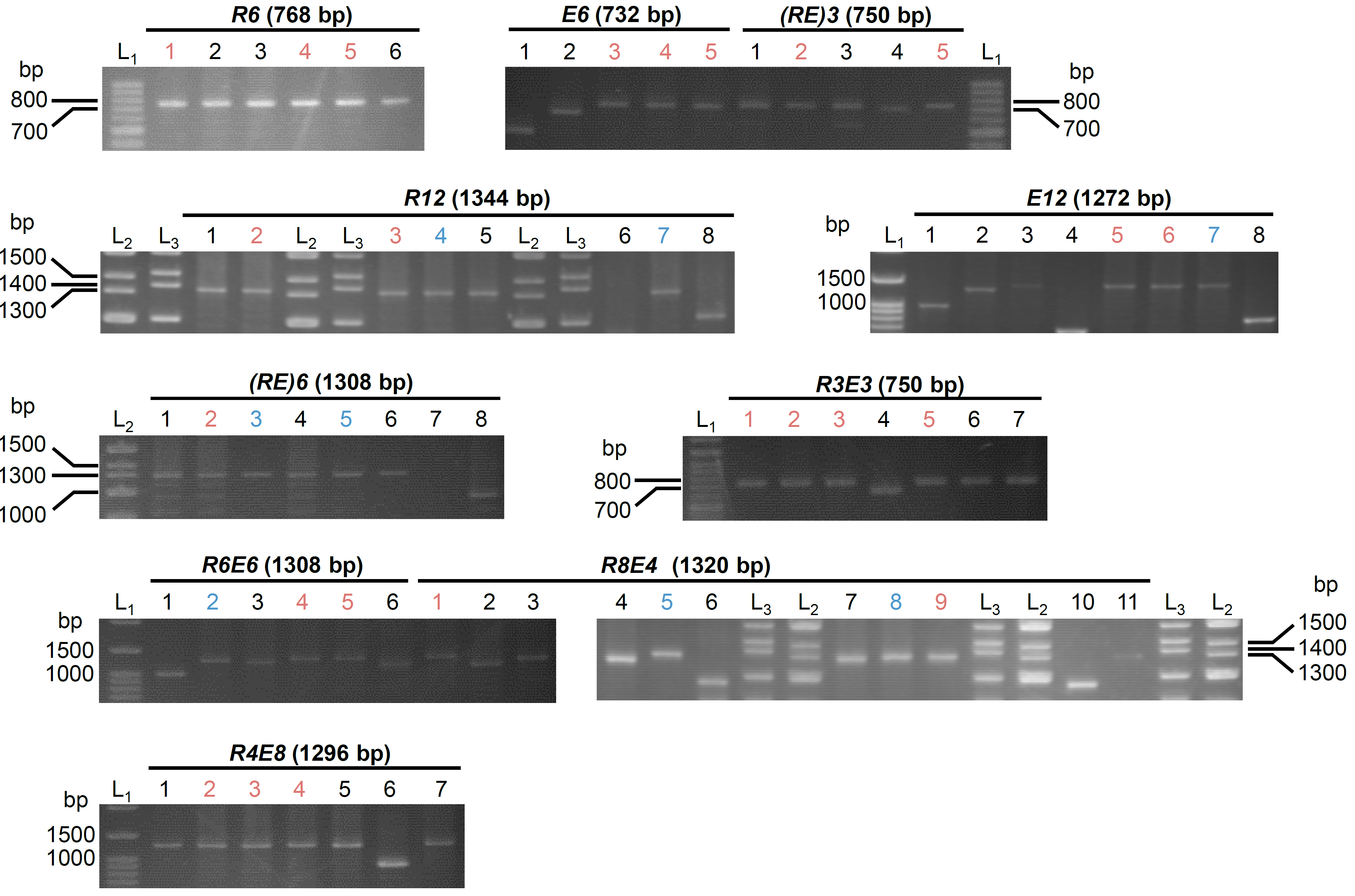


**Figure S2. Colony PCR results of transformants with various repetitive-sequence genes**

Transformants presumed to contain the gene of interest were selected using colony PCR. The size of the colony PCR products was confirmed by electrophoresis using a 1.5% agarose gel. [L_1_: 100 bp DNA ladder Plus (Nippon Genetics); L_2_: Gene ladder Wide 1 (Nippon Gene); L_3_: Wide-Range DNA Ladder (Takara Bio, Shiga, Japan)]. The number of bases in parentheses represents the theoretical length of the colony PCR product. For the transformants identified by red and blue font, the gene sequences were confirmed by Sanger DNA sequencing, which revealed that the red-font transformants had the correct repetitive sequence.


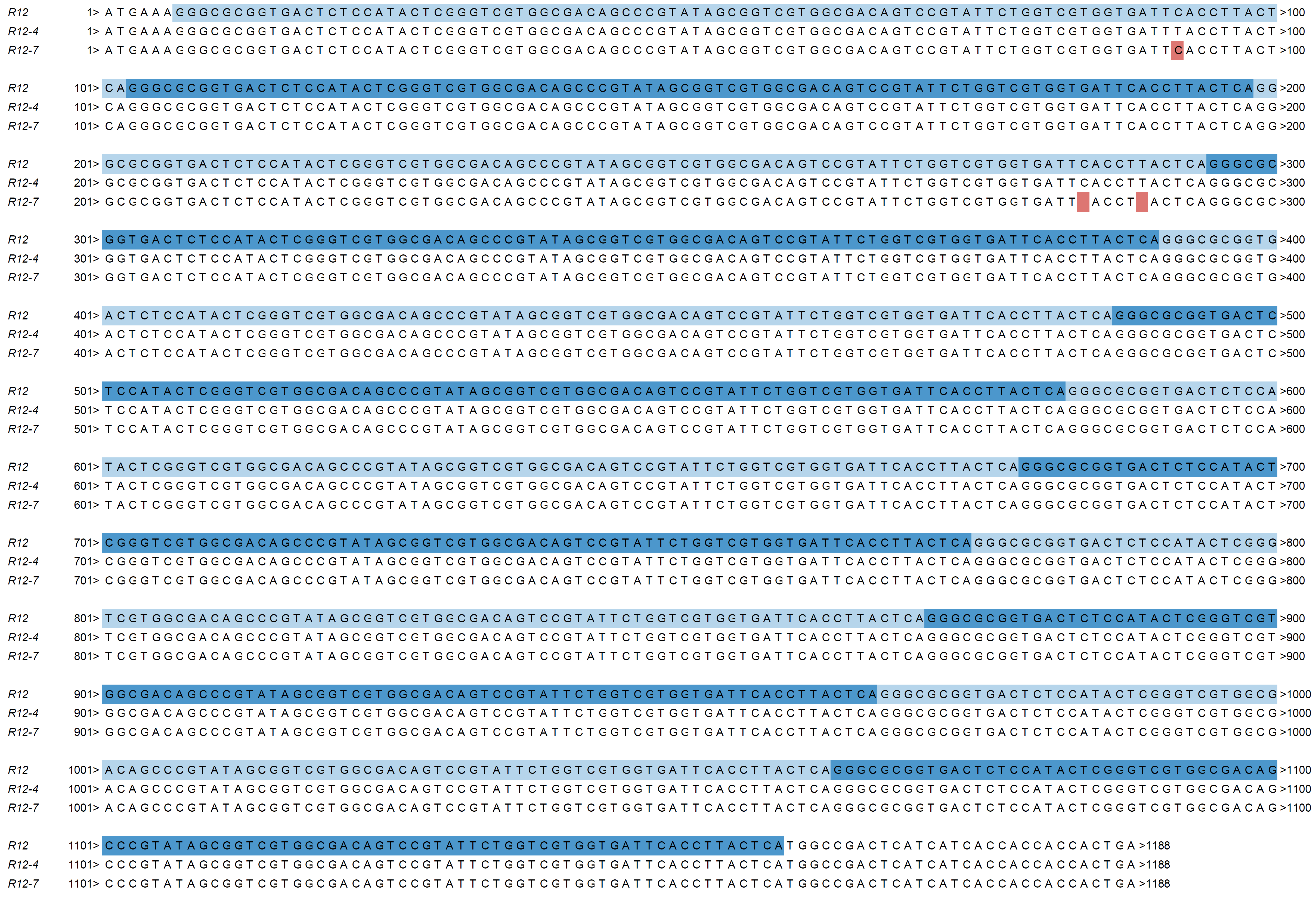


**Figure S3. Alignment of the correct open reading frame and the error nucleotide sequences of *R12***

Nucleotides marked in red differ from those present in the designed sequence. To easily identify repetitive sequences, odd repetitions are indicated in light blue and even repetitions in blue.


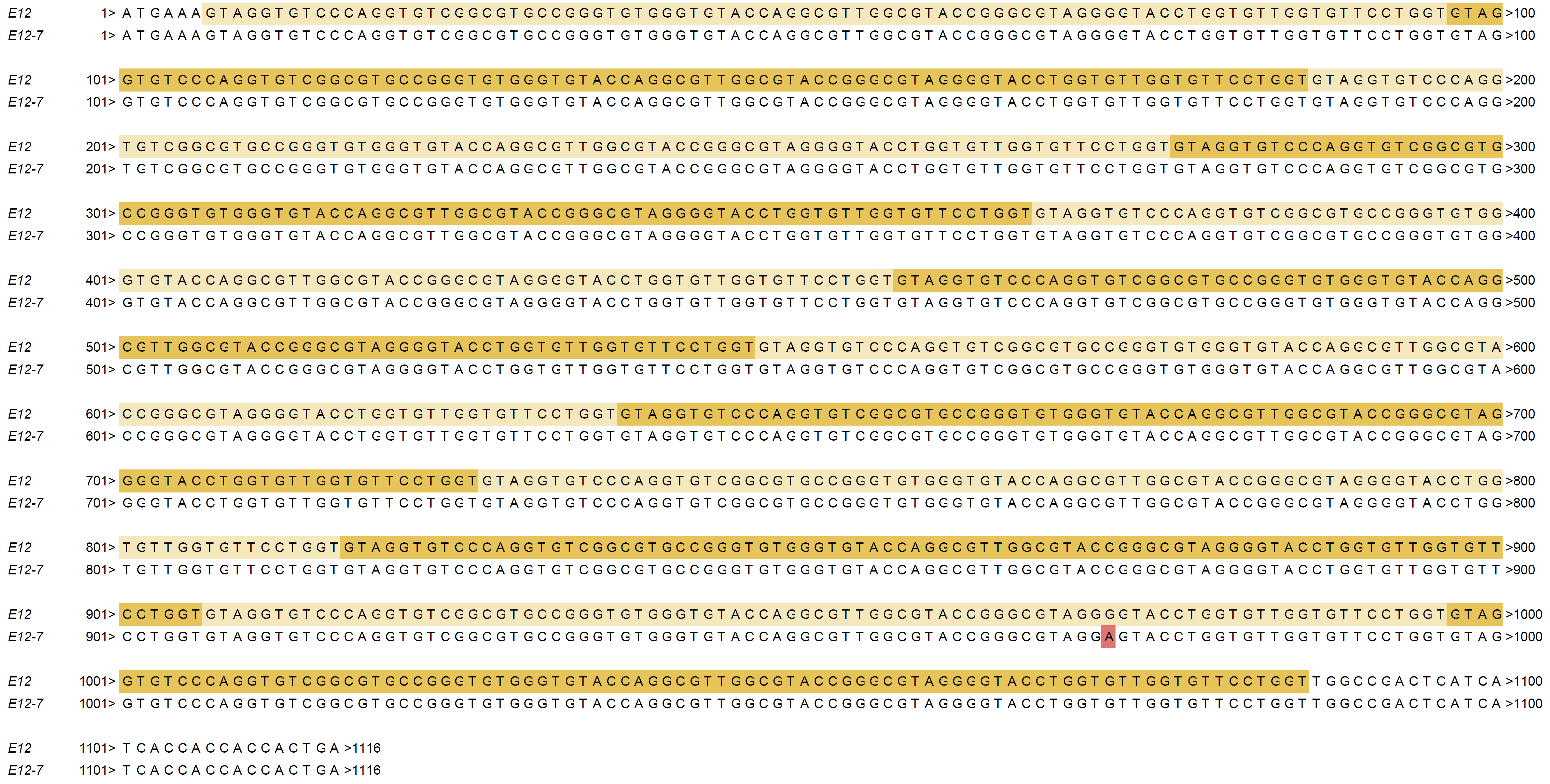


**Figure S4. Alignment of the correct open reading frame and the error nucleotide sequences of *E12***

Nucleotides marked in red differ from those present in the designed sequence. To easily identify repetitive sequences, odd repetitions are indicated in light yellow and even repetitions in yellow.


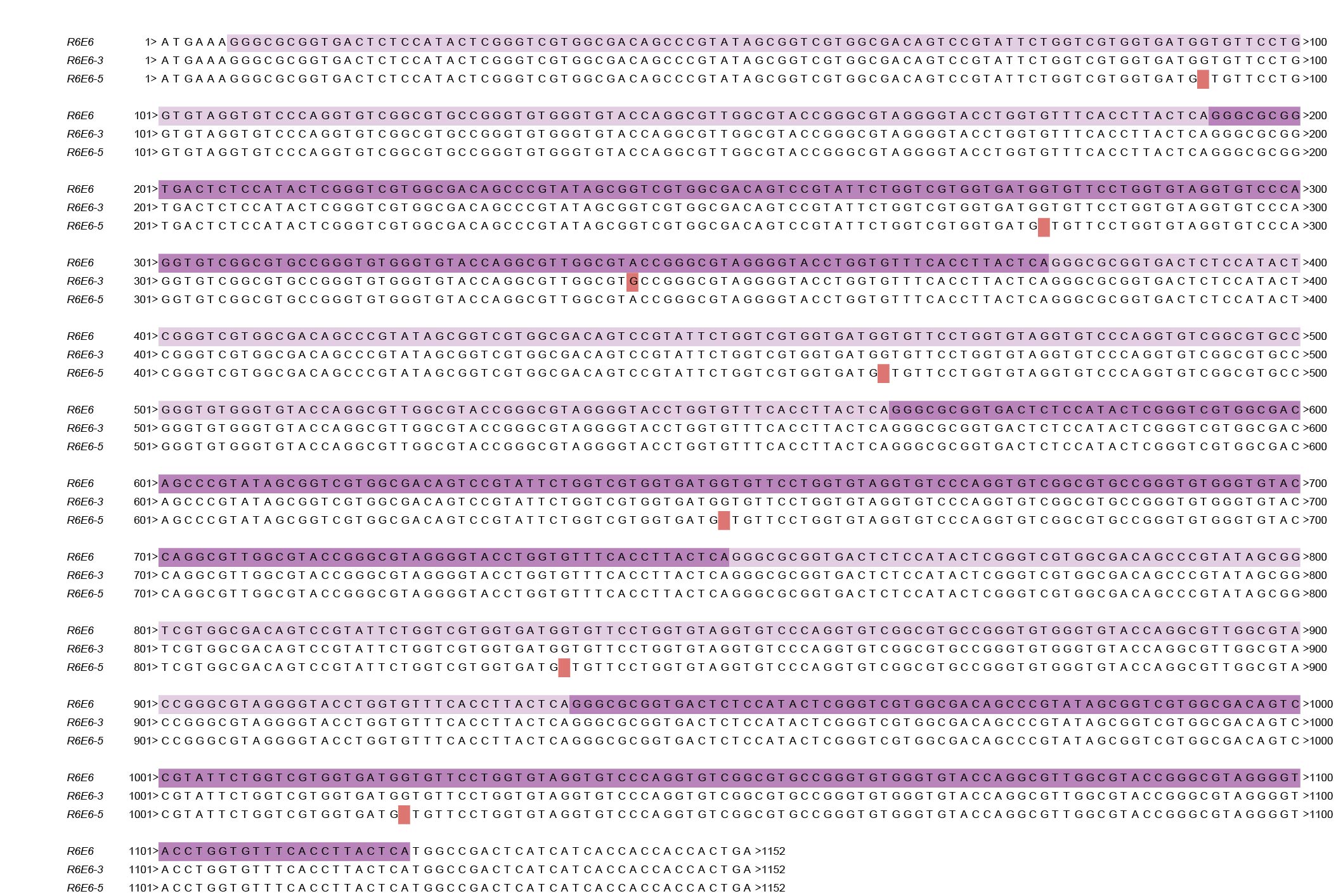


**Figure S5. Alignment of the correct open reading frame and the error nucleotide sequences of *(RE)6***

Nucleotides marked in red differ from those present in the designed sequence. To easily identify repetitive sequences, odd repetitions are indicated in light purple and even repetitions in purple. The gaps observed in *(RE)6*-5 can be attributed to an incomplete template.


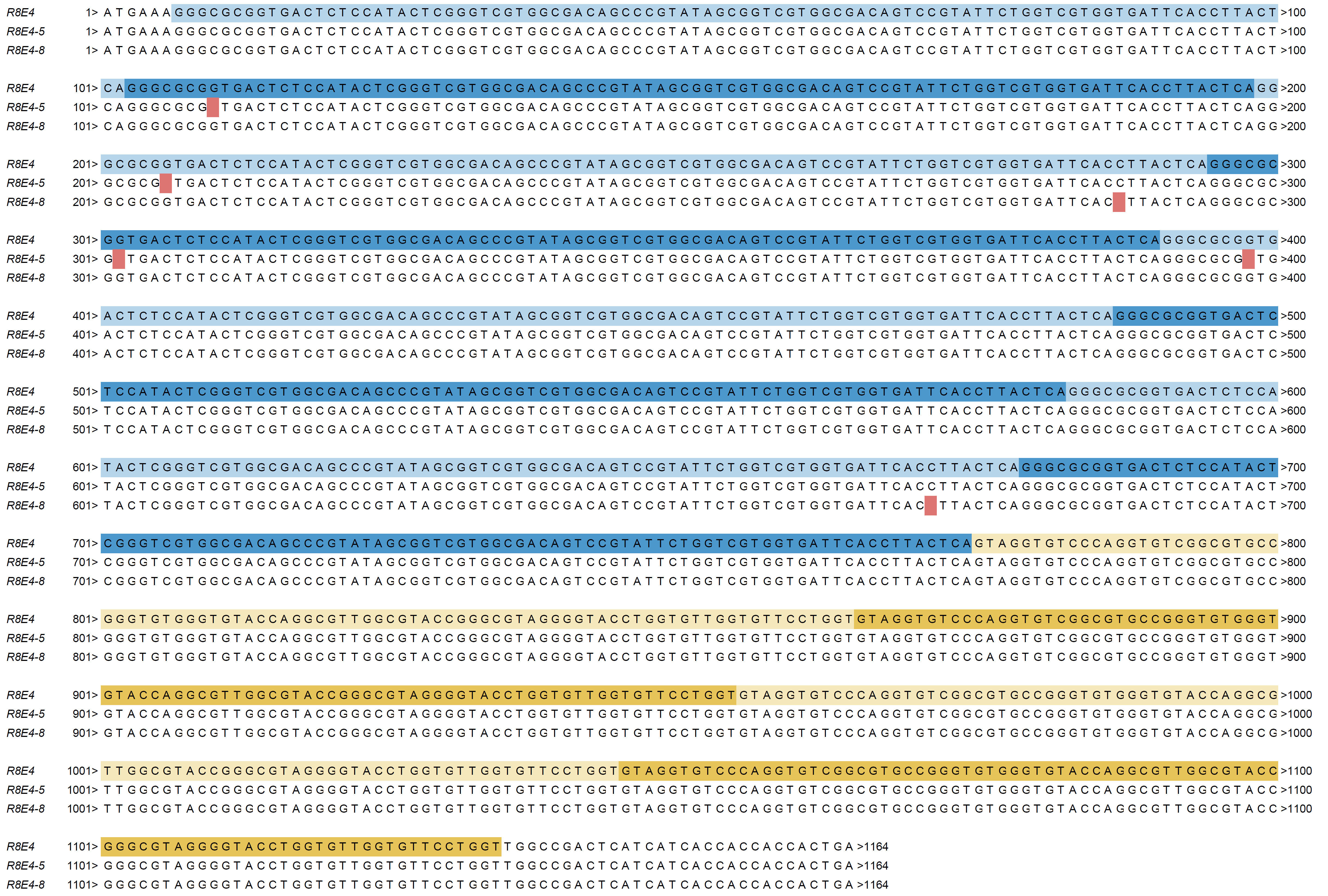


**Figure S6. Alignment of the correct open reading frame and the error nucleotide sequences of *R8E4***

Nucleotides marked in red differ from those present in the designed sequence. To easily identify repetitive sequences, odd repetitions of the R repeats are indicated in light blue and even repetitions in blue, and odd repetitions of the E repeats are indicated in light yellow and even repetitions in yellow. The gaps seen in *R8E4*-5 and *R8E4*-*8* can be attributed to an incomplete template.


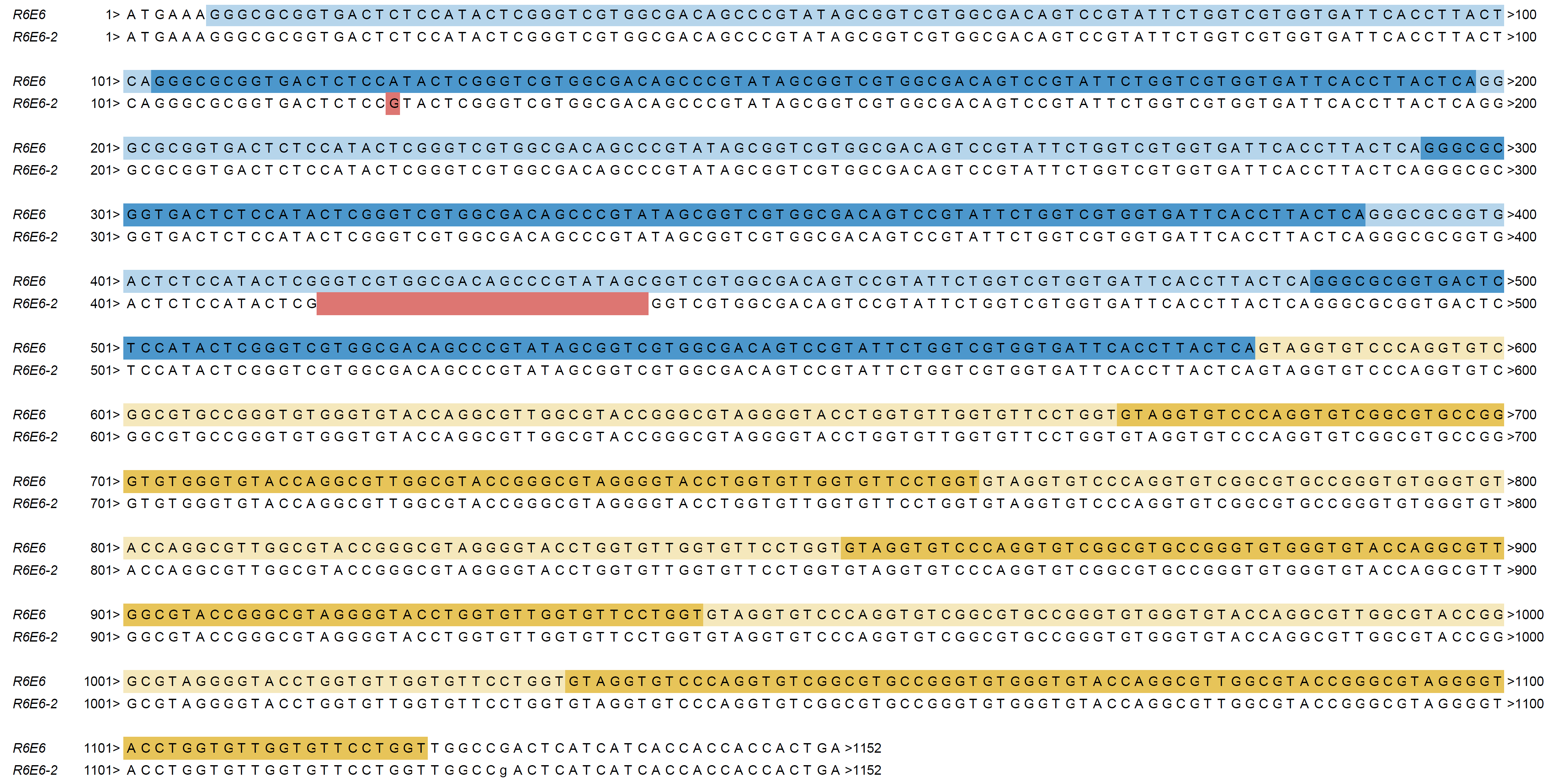


**Figure S7. Alignment of the correct open reading frame and the error nucleotide sequences of *R6E6***

Nucleotides marked in red differ from those present in the designed sequence. To easily identify repetitive sequences, odd repetitions of the R repeats are indicated in light blue and even repetitions in blue, and odd repetitions of the E repeats are indicated in light yellow and even repetitions in yellow. The continuous 24-base gap in *R6E6*-2 was assumed to be an error caused by secondary structure formation or cloning failure.

**Figure S8. Roadmaps for constructing the *E12* gene.**

The processes for constructing the target gene from oligo DNA are shown. For the combined method of concatemerization and PRe-RDL, because long genes were difficult to obtain via concatemerization, we performed PRe-RDL twice after obtaining the *E4* gene.

In CCS, a target value for *E12* was calculated using a program developed by the Chilkoti lab (https://chilkotilab.pratt.duke.edu/codon-scrambler) before creating a roadmap. Since the target value exceeded the reference value (2.47 × 10^6^), it was difficult to synthesize the gene in one step by PCR, and therefore, we synthesized the *E4* gene followed by the Golden Gate Assembly to synthesize the *E12* gene.

**Figure S9. Roadmaps for constructing the *R8E4*, *R6E6*, and *R4E8* genes.**

The processes for constructing target genes from oligo DNA are shown. Since OERCA alone cannot construct block copolymers, we assumed that PRe-RDL would be performed after constructing the block portion.


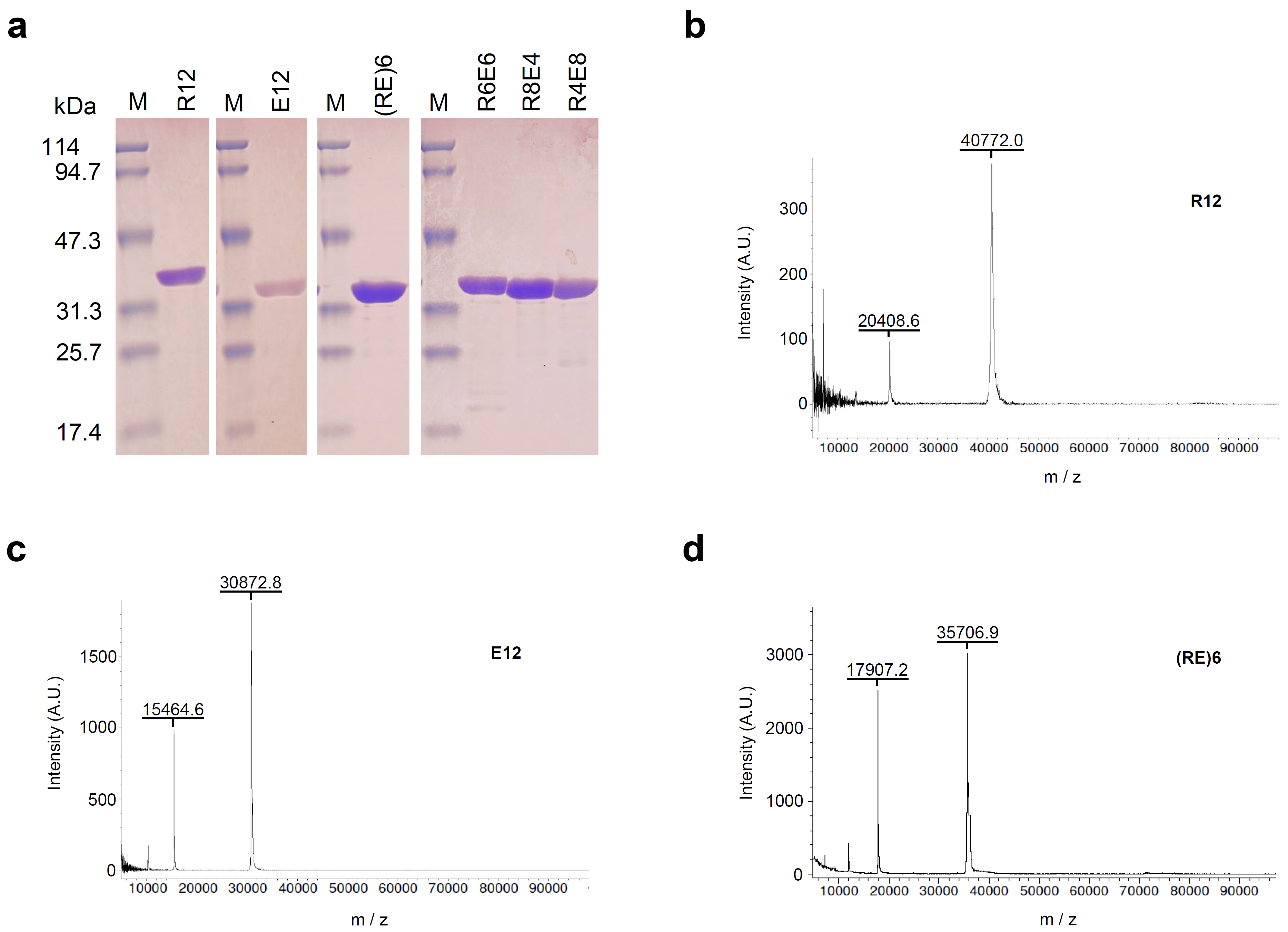


**Figure S10. Molecule sizes of the purified protein polymers**

(a) Lane M contains EzStandard PrestainBlue molecular-weight marker (ATTO, Tokyo, Japan). Sodium dodecyl-polyacrylamide gel electrophoresis (SDS-PAGE) results confirmed that R12, E12, (RE)6, R8E4, R6E6, and R4E8 were highly purified. Since, proline-rich protein polymers do not fully elongate even in the presence of SDS, their mobilities differ from the theoretical pattern. The slight difference in band position may be attributed to the effect of proline.^[1,2]^ (b-d) Sanger DNA sequencing confirmed the repeat numbers of R8E4, R6E6, and R4E8, but could not confirm those of R12, E12, and (RE)6. Therefore, their masses were measured via matrix-assisted laser desorption ionization time-of-flight mass spectrometry. The theoretical molecular weights of (b) R12, (c) E12, and (d) (RE)6 were 40817, 30949, and 35883, respectively; therefore, all three had masses within 0.5% of the theoretical values. This indicates that R12, E12, and (RE)6 were constructed as designed.


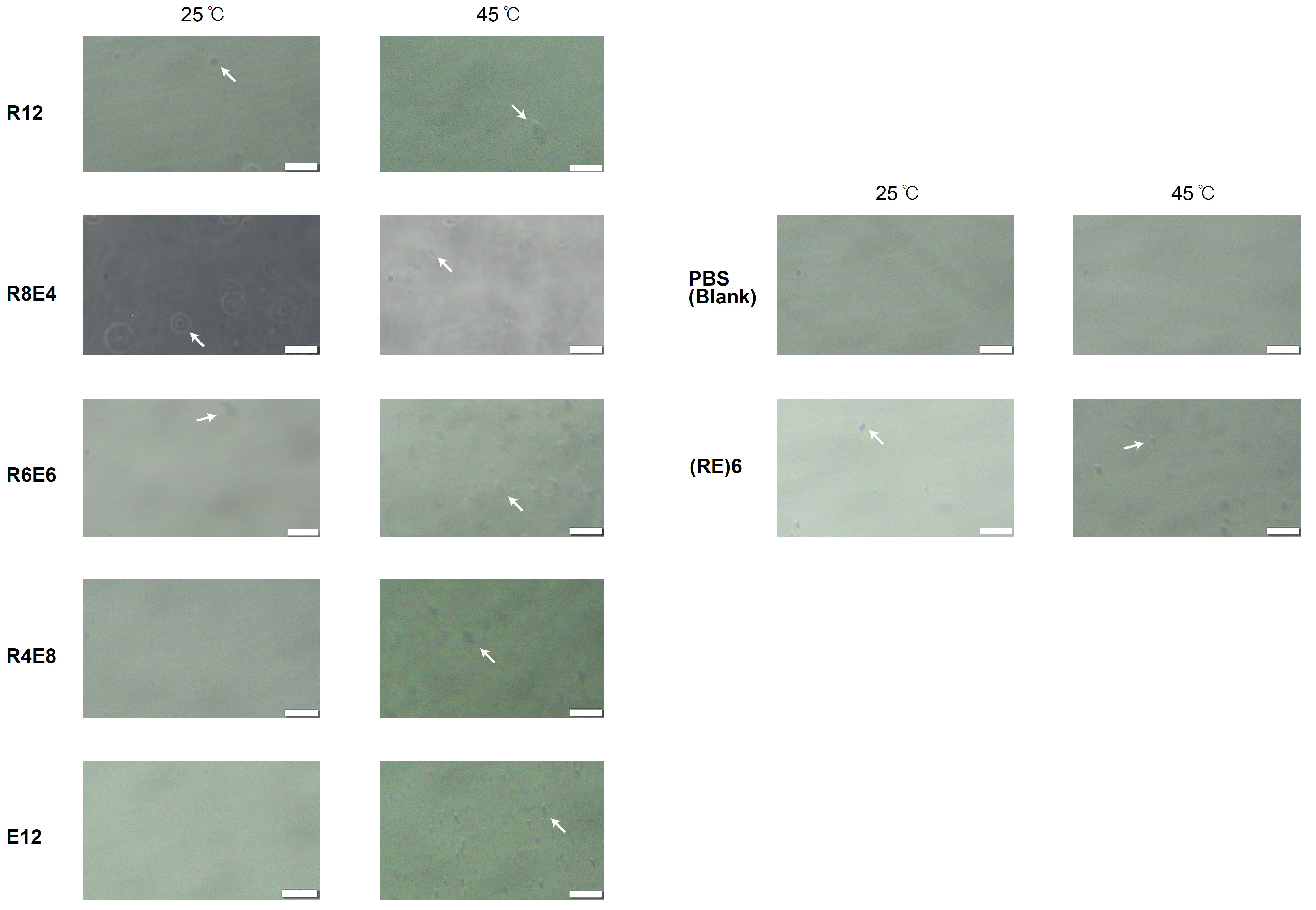


**Figure S11. Microscopic observation of the copolymer set prepared by SCRCA.**

Phase contrast microscopy images of PBS containing 2 μM polymer. The left and right images were taken at 25°C and 45°C, respectively. The arrows indicate polymer aggregates. Scale bar: 20 μm.

**Figure S12. Colony PCR results of the transformant library**

The transformants indicated using red and blue fonts were presumed to contain the target gene using colony PCR. The lengths of the colony PCR products were confirmed by electrophoresis using a 1.5% agarose gel (L: 100 bp DNA ladder Plus). The theoretical length is 732 bp. Sanger DNA sequencing revealed that the red transformants were E6 mutants. No analyzable sequence result was obtained from the No. 35 colony.


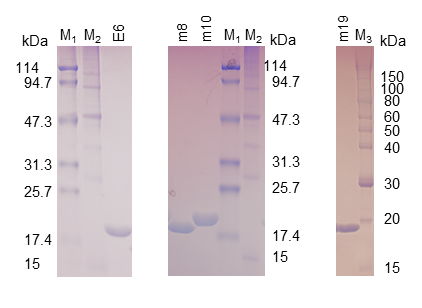


**Figure S13. SDS-PAGE images of the E6 mutants**

Lanes M_1_, M_2_, and M_3_ contain the EzStandard PrestainBlue molecular-weight marker (ATTO), Broad Range Protein Molecular Weight Markers (Promega, Madison, USA), and SIMASIMA Unstained Broad Range Protein Ladder (Cosmo Bio, Tokyo, Japan), respectively. No bands of contaminant proteins were observed, indicating that high-purity E6, m8, m10, and m19 were prepared.

**Figure S14. Colony PCR results of the transformants obtained for the second round**

Transformants presumed to contain the target gene were selected using colony PCR. The length of the colony PCR products was confirmed by electrophoresis using a 1.5% agarose gel (L: 100 bp DNA ladder Plus). The theoretical length is 732 bp. The transformants indicated in red and blue fonts were determined to contain inserted DNA fragments of similar length as the target repeat sequence. DNA sequencing revealed that the red-font transformants were m10 mutants. No analyzable sequence result was obtained from the no. 43 colony.


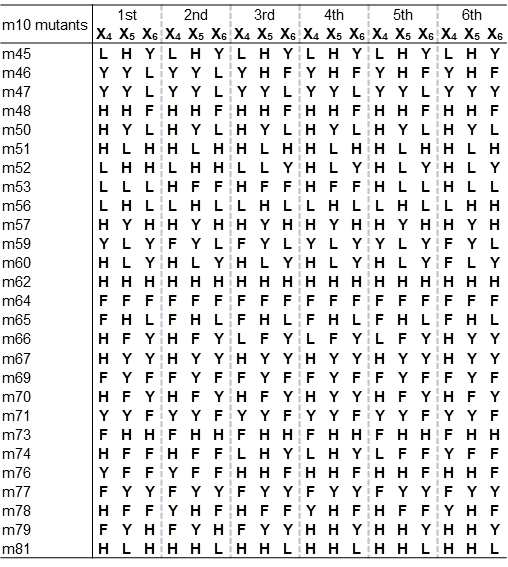


**Figure S15. Mutant library constructed for the second round**

For the 27 m10 mutants in the library, the amino acids at the X_4_, X_5_, and X_6_ positions are summarized. One of the four expected amino acids was introduced for all positions.

**Figure S16. SDS-PAGE images of the m10 mutants**

Lane M contains SIMASIMA Unstained Broad Range Protein Ladder (Cosmo Bio, Tokyo, Japan). No bands of contaminant proteins were observed, indicating that m48, m60, m62, m64, m65, m71, and m76 were of high purity.

**Table S1. Costs of oligo DNAs for synthesis of the *E12* gene.**

The length and number of oligos were determined based on the existing literature. Purification methods and oligo prices were based on information from the website of Eurofin Genomics Inc.

| Length (nt) | Option | Purification | Number | Cost ($) |
| --- | --- | --- | --- | --- |
|  |  |  |  |  |
| **SCRCA** |  |  |  |  |
| 90 | 5'-phosphorylation | HPLC | 1 | 118.3 |
| 39 |  | OPC | 1 | 13.8 |
| <35 |  | OPC | 1 | 6.6 |
| Total |  |  |  | **138.7** |
|  |  |  |  |  |
| **Concatemerization + PRe-RDL** | | |  |  |
| 90 |  | HPLC | 2 | 156.7 |
| Total |  |  |  | **156.7** |
|  |  |  |  |  |
| **OERCA** |  |  |  |  |
| 90 |  | HPLC | 1 | 78.3 |
| <35 |  | OPC | 2 | 13.2 |
| Total |  |  |  | **91.5** |
|  |  |  |  |  |
| **CCS** |  |  |  |  |
| 90 |  | HPLC | 8 | 626.7 |
| <35 |  | OPC | 8 | 52.8 |
| Total |  |  |  | **679.5** |
|  |  |  |  |  |

**Table S2. Costs of oligo DNAs for synthesizing *R4E8*, *R6E6*, and *R8E4* genes.**

The length and number of oligos were determined based on the existing literature. Purification methods and oligo prices were based on information from the website of Eurofins Genomics Inc.

| Length (nt) | Option | Purification | Number | Cost ($) |
| --- | --- | --- | --- | --- |
|  |  |  |  |  |
| **SCRCA** |  |  |  |  |
| 96 | 5'-phosphorylation | HPLC | 1 | 121.3 |
| 90 | 5'-phosphorylation | HPLC | 1 | 118.3 |
| 39 |  | OPC | 1 | 13.8 |
| <35 |  | OPC | 3 | 19.8 |
| Total |  |  |  | **273.2** |
|  |  |  |  |  |
| **Concatemerization + PRe-RDL** | |  |  |  |
| 96 |  | HPLC | 2 | 162.7 |
| 90 |  | HPLC | 2 | 156.7 |
| Total |  |  |  | **319.3** |
|  |  |  |  |  |
| **OERCA + PRe-RDL** | |  |  |  |
| 96 |  | HPLC | 1 | 81.3 |
| 90 |  | HPLC | 1 | 78.3 |
| <35 |  | OPC | 4 | 26.4 |
| Total |  |  |  | **186.1** |
|  |  |  |  |  |
| **CCS** |  |  |  |  |
| 90 |  | HPLC | 28 | 2193.3 |
| <35 |  | OPC | 20 | 132.0 |
| Total |  |  |  | **2325.3** |
|  |  |  |  |  |

**Table S3. Reagent costs for each process.**

Reagent prices were surveyed in April 2024. Prices were converted to USD with a currency conversion rate of 150 yen per USD.

| Process | Reagent | Catalog No. | Company | Cost ($) |
| --- | --- | --- | --- | --- |
|  |  |  |  |  |
| **RCA for seamless cloning** |  |  |  |  |
| Cyclization | T4 DNA ligase | M0202S | NEB | 1.5 |
| RCA | Bst DNA polymerase large fragment | M0275S | NEB | 1.0 |
|  | 25 mM dNTP mixture | 312-07271 | Nippon Gene | 1.0 |
| Total |  |  |  | **3.5** |
|  |  |  |  |  |
| **Seamless cloning** | In-Fusion® HD Cloning Kit | 639648 | Takara Bio | **16.7** |
|  |  |  |  |  |
| **Concatemerization** | T4 DNA ligase | M0202S | NEB | **1.5** |
|  |  |  |  |  |
| **PRe-RDL** |  |  |  |  |
| Restriction enzyme treatment | AcuI | R0641S | NEB | 2.9 |
|  | BglI | R0143S | NEB | 3.0 |
|  | BseRI | R0581S | NEB | 3.9 |
| Total |  |  |  | **9.8** |
|  |  |  |  |  |
| **Ligation** | T4 DNA ligase | M0202S | NEB | **1.5** |
|  |  |  |  |  |
| **OERCA** |  |  |  |  |
| Cyclization | CircLigase ssDNA Ligase | CL4111K | AR BROWN | 83.3 |
| Exonuclease treatment | Exonuclease I | 2650A | Takara Bio | 3.1 |
| RCA | PfuUltra™ II Fusion HS DNA polymerase | 600670 | Agilent Technologies | 9.7 |
|  | 25 mM dNTP mixture | 312-07271 | Nippon Gene | 16.7 |
| Ligation | T4 DNA ligase | M0202S | NEB | 0.0 |
| Total |  |  |  | **112.7** |
|  |  |  |  |  |
| **PCR** | Herculase II Fusion DNA Polymerase | 600675 | Agilent Technologies | **1.0** |
|  |  |  |  |  |
| **Golden gate** |  |  |  |  |
| Reaction | BsaI-HF®v2 | R3733S | NEB | 1.7 |
|  | T7 ligase | M0318S | NEB | 0.7 |
| Purification | PlasmidSafe DNAse | E3101K | AR BROWN | 1.7 |
| Total |  |  |  | **4.1** |
|  |  |  |  |  |

**Table S4. Reagent costs for *E12* gene synthesis.**

The reagent cost was calculated by multiplying the reagent cost for each step (Table S3) by the number of samples based on the roadmap (Figure S8).

| Process | Sample number | Cost ($) |
| --- | --- | --- |
|  |  |  |
| **SCRCA** |  |  |
| RCA for seamless cloning | 1 | 3.5 |
| Seamless cloning | 1 | 16.7 |
| Total |  | **20.2** |
|  |  |  |
| **Concatemerization + PRe-RDL** |  |  |
| Concatemerization | 1 | 1.5 |
| Restriction enzyme treatment | 2 | 19.6 |
| Ligation | 2 | 3.0 |
| Total |  | **24.1** |
|  |  |  |
| **OERCA** |  |  |
| OERCA | 1 | 112.7 |
| Total |  | **112.7** |
|  |  |  |
| **CCS** |  |  |
| PCR | 1 | 1.0 |
| Golden gate | 1 | 4.1 |
| Total |  | **5.1** |
|  |  |  |

**Table 5S. Reagent costs for synthesizing *R4E8*, *R6E6*, and *R8E4* genes.**

The reagent cost was calculated by multiplying the reagent cost for each step (Table S3) by the number of samples based on the roadmap (Figure S9).

| Process | Sample number | Cost ($) |
| --- | --- | --- |
|  |  |  |
| **SCRCA** |  |  |
| RCA for seamless cloning | 1 | 3.5 |
| Seamless cloning | 3 | 50.0 |
| Total |  | **53.5** |
|  |  |  |
| **Concatemerization + PRe-RDL** |  |  |
| Concatemerization | 2 | 3.0 |
| Restriction enzyme treatment | 8 | 78.3 |
| Ligation | 7 | 10.6 |
| Total |  | **91.9** |
|  |  |  |
| **OERCA + PRe-RDL** |  |  |
| OERCA | 2 | 225.5 |
| PRe-RDL | 6 | 58.7 |
| Ligation | 9 | 13.7 |
| Total |  | **284.2** |
|  |  |  |
| **CCS** |  |  |
| PCR | 4 | 4.1 |
| Golden gate | 3 | 12.3 |
| Total |  | **16.4** |
|  |  |  |

**Table S6. Screening costs for the construction of the *E12* gene.**

It was assumed that Promega Go taq green mix (catalog no. M7122) would be used for colony PCR. It costs $1 per colony. DNA sequencing was assumed to be performed by Premix 2 of Fasmac Co., Ltd. (<https://fasmacfasmac.co.jp/dnaseq/premix2>), which costs $36.3 per gene.

| Process | Sample number | Cost ($) |
| --- | --- | --- |
|  |  |  |
| **SCRCA** |  |  |
| Colony PCR | 8 | 7.7 |
| Sangar DNA sequencing | 1 | 36.3 |
| Total |  | **44.0** |
|  |  |  |
| **Concatemerization + PRe-RDL** |  |  |
| Colony PCR | 116 | 112.1 |
| Sanger DNA sequencing | 3 | 108.9 |
| Total |  | **221.0** |
|  |  |  |
| **OERCA** |  |  |
| Colony PCR | 280 | 270.7 |
| Sanger DNA sequencing | 1 | 36.3 |
| Total |  | **307.0** |
|  |  |  |
| **CCS** |  |  |
| Colony PCR | 16 | 15.5 |
| Sanger DNA sequencing | 2 | 72.6 |
| Total |  | **88.1** |
|  |  |  |

**Table S7. Screening costs for the construction of the *R4E8*, *R6E6*, and *R8E4* genes.**

It was assumed that Promega Go taq green mix (catalog no. M7122) was used for colony PCR, which costs $1 per colony. DNA sequencing was assumed to be performed by Premix 2 of Fasmac Co., Ltd. (<https://fasmacfasmac.co.jp/dnaseq/premix2>), which costs $36.3 per gene.

| Process | Sample number | Cost ($) |
| --- | --- | --- |
|  |  |  |
| **SCRCA** |  |  |
| Colony PCR | 24 | 23.2 |
| Sangar DNA sequencing | 3 | 108.9 |
| Total |  | **132.1** |
|  |  |  |
| **Concatemerization + PRe-RDL** |  |  |
| Colony PCR | 264 | 255.2 |
| Sangar DNA sequencing | 11 | 399.3 |
| Total |  | **654.5** |
|  |  |  |
| **OERCA + PRe-RDL** |  |  |
| Colony PCR | 584 | 564.5 |
| Sangar DNA sequencing | 9 | 326.7 |
| Total |  | **891.2** |
|  |  |  |
| **CCS** |  |  |
| Colony PCR | 56 | 54.1 |
| Sangar DNA sequencing | 7 | 254.1 |
| Total |  | **308.2** |
|  |  |  |

**Table S8. Combinations of phosphorylated ssDNA oligos and primer sets for isothermal amplification.**

The underlined nucleotides represent the annealing sites of the reverse primers, and double-underlined nucleotides represent those of the forward primers. The RE ring was prepared using a split oligo DNA (ACCAGGAACACCATCACCACGACC) to connect two 5′-phosphorylated ssDNAs. N, D, Y, and W represent mixed bases (N: A, C, G, or T; D: G, A, or T; Y: C or T; and W: A or T).

| Template name | 5’ phosphorylated ssDNA | Base length | Forward primer | Reverse primer |
| --- | --- | --- | --- | --- |
| R | TCACCTTACTCAGGGCGCGGTGACTCTCCATACTCGGGTCGTGGCGACAGCCCGTATAGCGGTCGTGGCGACAGTCCGTATTCTGGTCGTGGTGAT | 96 | GATATACATATGAAAGGGCGCGGTGACTCTCC | ATGATGAGTCGGCCATGAGTAAGGTGAATCACCACGACC |
| E | GGTGTTCCTGGTGTAGGTGTCCCAGGTGTCGGCGTGCCGGGTGTGGGTGTACCAGGCGTTGGCGTACCGGGCGTAGGGGTACCTGGTGTT | 90 | GATATACATATGAAAGTAGGTGTCCCAGGTGTCGG | ATGATGAGTCGGCCAACCAGGAACACCAACACCAGGTAC |
| RE | TCACCTTACTCAGGGCGCGGTGACTCTCCATACTCGGGTCGTGGCGACAGCCCGTATAGCGGTCGTGGCGACAGTCCGTATTCTGGTCGTGGTGATGGTGTTCCTGGTGTAGGTGTCCCAGGTGTCGGCGTGCCGGGTGTGGGTGTACCAGGCGTTGGCGTACCGGGCGTAGGGGTACCTGGTGTT | 186 | GATATACATATGAAAGGGCGCGGTGACTCTCC | ATGATGAGTCGGCCATGAGTAAGGTGAAACACCAGGTAC |
| E-3NDT | GGTGTTCCTGGTGTAGGTGTCCCAGGTGTCGGCGTGCCGGGTNDTGGTGTACCAGGCNDTGGCGTACCGGGCNDTGGGGTACCTGGTGTT | 90 | GATATACATATGAAAGTAGGTGTCCCAGGTGTCGG | ATGATGAGTCGGCCAACCAGGAACACCAACACCAGGTAC |
| m10-3YWY | GGTGTTCCTGGTCACGGTGTCCCAGGTATTGGCGTGCCGGGTYWYGGTGTACCAGGCYWYGGCGTACCGGGCYWYGGGGTACCTGGTCAC | 90 | GATATACATATGAAACACGGTGTCCCAGGTATTGG | ATGATGAGTCGGCCAACCAGGAACACCGTGACCAGGTAC |

**Parameters used in NGS analysis**

### ① Adapter Trimming ###

<programs>

cutadapt:1.1

<options>

R1 reads: cutadapt --match-read-wildcards -O 1 -e 0.2 -a CTGTCTCTTATACACATCTCCGAGCCCACGAGAC

R2 reads: cutadapt --match-read-wildcards -O 1 -e 0.2 -a CTGTCTCTTATACACATCTGACGCTGCCGACGA

<input>

R1 reads: *R1*_fastq.gz (R1 raw_read_data)

R2 reads: *R2*_fastq.gz (R2 raw_read_data)

<output>

Adapter Trimmed Reads

<usage>

cutadapt

-a "Sequence of an adapter that was ligated to the 3' end"

--match-read-wildcards "Allow 'N's in the read as matches to the adapter"

-O "Minimum overlap length"

-e "Maximum allowed error rate"

### ② Quality Trimming ###

<programs>

Trimmomatic:0.32

<options>

trimmomatic -phred33 LEADING:0 TRAILING:0 SLIDINGWINDOW:20:20 MINLEN:50

<input>

Adapter Trimmed Reads

<output>

Adapter and Quality Trimmed Reads

<usage>

Trimmomatic

-phred33 "Fastq base quality encoding: phred33"

LEADING:<quality> "<quality> Specifies the minimum quality required to keep a base"

TRAILING:<quality> "<quality> Specifies the minimum quality required to keep a base"

SLIDINGWINDOW:<windowSize>:<requiredQuality> "<windowSize> Specifies the number of bases to average across, <requiredQuality> Specifies the average quality required"

MINLEN:<length> "Specifies the minimum length of reads to be kept"

**References**

[1] C. Scheller, F. Krebs, R. Wiesner, H. Wätzig, I. Oltmann-Norden, *Electrophoresis* **2021**, *42*, 1521.

[2] C.M. Elvin, A.G. Carr, M.G. Huson, J.M. Maxwell, R.D. Pearson, T. Vuocolo, N.E.

Liyou, D.C.C. Wong, D.J. Merritt, N.E. Dixon, *Nature* **2005**, *437*, 999.
